# Opportunistic dose amplification for proton and carbon ion therapy via capture of internally generated thermal neutrons

**DOI:** 10.1038/s41598-018-34643-w

**Published:** 2018-11-02

**Authors:** Mitra Safavi-Naeini, Andrew Chacon, Susanna Guatelli, Daniel R. Franklin, Keith Bambery, Marie-Claude Gregoire, Anatoly Rosenfeld

**Affiliations:** 1 Australian Nuclear Science and Technology Organisation (ANSTO), Sydney, Australia; 2 Centre for Medical Radiation Physics, University of Wollongong, Sydney, Australia; 3 Faculty of Engineering & IT, University of Technology Sydney, Sydney, Australia

## Abstract

This paper presents Neutron Capture Enhanced Particle Therapy (NCEPT), a method for enhancing the radiation dose delivered to a tumour relative to surrounding healthy tissues during proton and carbon ion therapy by capturing thermal neutrons produced inside the treatment volume during irradiation. NCEPT utilises extant and in-development boron-10 and gadolinium-157-based drugs from the related field of neutron capture therapy. Using Monte Carlo simulations, we demonstrate that a typical proton or carbon ion therapy treatment plan generates an approximately uniform thermal neutron field within the target volume, centred around the beam path. The tissue concentrations of neutron capture agents required to obtain an arbitrary 10% increase in biological effective dose are estimated for realistic treatment plans, and compared to concentrations previously reported in the literature. We conclude that the proposed method is theoretically feasible, and can provide a worthwhile improvement in the dose delivered to the tumour relative to healthy tissue with readily achievable concentrations of neutron capture enhancement drugs.

## Introduction

The principal aim of all forms of radiation therapy is to deliver the maximum therapeutic radiation dose to the target, while sparing surrounding healthy tissue. One of the greatest challenges of radiotherapy is to minimise its latent effects - including the risk of secondary cancer, which can occur anywhere from five years to many decades post-treatment^1–4^. The objective is to minimise normal tissue complication probability (NTCP) - which includes the probability of developing treatment-induced cancers - by maximising the conformity of the delivered dose to the target volume^4,5^. Technological advancements in radiotherapy such as intensity modulated radiotherapy, image-guided radiotherapy and particle therapies have enabled more accurate and selective targeting of the tumour^6^. Many of these treatments can also be enhanced by the use of tumour-specific radiosensitisers, which increase the contrast between the biological dose delivered to the tumour and healthy tissues^7^.

Particle therapy is a form of radiotherapy in which a beam of highly energetic protons or heavy ions (most commonly carbon) is used to deliver a therapeutic radiation dose to a treatment region. Monoenergetic beams of protons and heavy ions exhibit a very well-defined Bragg peak, depositing most of their kinetic energy within a narrow energy-dependent depth range. By tuning the beam energy, this property enables the delivery of a highly conformal radiation dose distribution to the target. Particle therapy is therefore ideal for treatment of deep tissues, since it results in a lower dose to healthy tissues in front of or behind the tumour compared to photon and electron beams^6,8,9^.

During particle therapy, most of the primary particles in the beam deposit their kinetic energy through multiple electromagnetic interactions with the target. However, a fraction of these particles will undergo nonelastic collisions with nuclei in the target. This results in the production of a range of nuclear fragments at the target site, including short-range, high-LET charged particles, and a mixture of fast and thermal neutrons. Due to scattering within the target tissue, the thermal neutrons are emitted nearly isotropically from the point of collision, and they deposit their energy in the region surrounding the path of the incident ion beam via a succession of elastic and inelastic collisions^10,11^. The thermal neutrons irradiate both target and non-target tissues indiscriminately, and they deposit a fraction of the beam’s kinetic energy outside of the target volume^9^. Such interactions are typically regarded as a nuisance when they occur outside of the treatment region, since their existence undermines one of the main advantages of particle therapy - the large peak-to-entrance-plateau dose ratio. It is also important to emphasise that these thermal neutrons are generated *internally* - in the vicinity of the target area inside the patient. This is quite distinct from the neutrons which result from contamination of the beam due to collisions between the beam and beam modifying devices.

Light water, the principal constituent of human tissue, has a moderate thermal neutron cross-section (0.335 barns^12^), which can be greatly increased by the introduction of agents containing isotopes such as ^10^B or ^157^Gd with very high neutron cross sections (3838 and 254000 barns, respectively^13,14^). While nonelastic thermal neutron interactions with water primarily result in hydrogen capture of the neutron and the release of a high-energy gamma photon, nonelastic thermal neutron interactions with ^10^B and ^157^Gd result in the production of energetic charged particles with high relative biological effectiveness - the basic operating principle of neutron capture therapy (NCT)^15,16^. Our central hypothesis is that if a sufficient thermal neutron fluence is generated during heavy ion therapy, it can be exploited therapeutically via the administration of a suitable non-toxic neutron capture agent containing ^10^B or ^157^Gd, preferentially absorbed by the tumour at an elevated concentration compared to the surrounding normal tissue as in conventional NCT. This combined therapeutic modality is denoted *neutron capture enhanced particle therapy* (NCEPT). To validate our hypothesis, and demonstrate the feasibility of NCEPT, it is necessary to determine the concentrations of neutron capture agents which would be required to achieve a therapeutically significant boost to tumour dose during a typical proton or carbon ion treatment plan.

In conventional neutron capture therapy, the biological dose due to the presence of the capture agent depends on the physical dose (which, in turn, depends on the concentration of neutron capture agent), together with the relative biological effectiveness (RBE) of the secondary particles as determined by the specific neutron capture agent (NCA). The latter factor varies significantly between different cell types and context (i.e., *in vitro* vs. *in vivo*); it is also specific to each specific neutron capture agent. For this work, only values reported for *in vivo* studies are relevant. In BNCT literature this compound-specific RBE factor is commonly referred to as *compound biological effectiveness* (CBE), however most researchers working with gadolinium simply refer to it as RBE. For consistency we refer to it henceforth as CBE for both boron and gadolinium.

In the case of ^10^B, the capture mechanism results in the production of several high LET products^17^:

1 $$\,{}^{10}{\rm{B}}+{n}_{th}\to {[{}^{11}{\rm{B}}]}^{\ast }\to \alpha +{}^{7}{\rm{L}}{\rm{i}}+\gamma \mathrm{(2.31}\,{\rm{MeV}})$$

Both the alpha particles and the lithium ions produce closely spaced ionisations in the immediate vicinity of the reaction, with a range of approximately 5–9 *μ*m; this is approximately the diameter of the target cells^18,19^.

For the most widely used ^10^B-based neutron capture agent, ^10^B-4-borono-L-phenylalanine (^10^B-BPA), CBE values of 3.6–3.8 and 0.9–1.3 have been reported for brain tumour cells and normal tissues, respectively, with tumour to healthy tissue concentration ratios between 5:1 and 8:1^16,20–23^. An alternative capture agent, borocaptate sodium (BSH), has shown potential for NCT applications; the reported range of CBE is between 1.2 and 2.3 in brain tumours and 0.37 to 0.5 in normal tissues, although the uptake concentration ratio tends to be much lower than for BPA (1.2–3.5 in the brain)^22,24,25^. The specific values differ for other target tissues, with higher values of CBE reported for liver tumours for both agents (tumour:liver CBE values of 9.94/4.25 and 4.22/0.94, and concentration ratios of 2.8/0.3 for BPA and BSH, respectively)^26^.

The ^157^Gd neutron capture reaction follows a somewhat different path, and results in the production of an excited ^158^Gd nucleus and a high-energy gamma ray:

2 $${}^{157}{\rm{Gd}}+{n}_{th}\to {{[}^{158}{\rm{Gd}}]}^{\ast }\to {}^{158}{\rm{Gd}}+\gamma +7.94\,{\rm{MeV}}$$

Upon relaxation of the excited state, internal conversion (IC) and low-energy Auger electrons are produced, the latter responsible for the majority of the useful therapeutic effects. Classified as a high-LET radiation, Auger electrons travel only a very short distance (a few nanometers in tissue) before depositing their kinetic energy, making them very effective if the source is concentrated in immediate vicinity of a DNA molecule or vital organelles (such as mitochondria)^27–32^. A yield of 5 Auger electrons, 1.8 *γ* photons and 0.69 IC electrons and 1.0 recoil nucleus has been estimated for the thermal neutron capture reaction^33,34^.

A number of studies have utilised Monte Carlo simulations to quantify the macroscopic, microscopic and nanoscopic components of the dose delivered by Auger-emitting isotopes in order to estimate their corresponding CBE. Humm *et al*. conducted a review of dosimetry of Auger electrons at DNA, subcellular and organ level, and recommended a CBE of 10 when the emitter is electrostatically bound to the DNA of the nucleus^35^. Fairlie performed a review of the radiation-weighted factors and RBEs of Auger-emitting isotopes, and recommended the adoption of CBEs of 5 and 20 for emitters of multiple Auger electrons incorporated within cells and DNA, respectively^36^. Cerullo *et al*. estimated the lineal energy distribution of Auger electrons within a 6 nm diameter cylinder (representing a DNA fragment) using the PENELOPE Monte Carlo simulation code, and obtained CBEs of 12.6, 6 and 1.5 where electrons are localised inside, on the surface and outside of the cylinder, respectively^31^.

^157^Gd is of great interest for neutron capture therapy due to its extremely high thermal neutron cross-section - the highest of any stable isotope. While free Gd^3+^ ion is highly toxic to organisms both *in vitro* and *in vivo*, chelated Gd^3+^ compounds can be used safely due to their physiological stability^37^. Very high cellular concentrations of gadolinium can be achieved *in vitro* without significant cytotoxicity (of the order of several thousand ppm)^38,39^. While gadolinium contrast agents such as Gd-DOTA and Gd-DTPA are approved for use in humans diagnostically, neither accumulates to significant concentration within the cell nucleus^40^. Amongst the experimental gadolinium compounds, motexafin-gadolinium (MGd) been proposed as a potential candidate for GdNCT^37^. It is a tumour-specific radiosensitiser, and its combined use with whole-brain radiation therapy has reached Phase III clinical trials^41^. With a 70:1 tumour to healthy tissue uptake ratio, prolonged retention of gadolinium *in vitro* (up to 2 months) and 90% uptake in glioblastoma cell nuclei, it is a promising candidate for use in NCT^37,42–44^. Recent efforts towards the development of DNA and mitochondria-targeting gadolinium agents has resulted in a number of promising agents. Morrison *et al*. have reported on the development of a tumour-cell selective mitochondrial agent designed for NCT applications, with cellular concentrations of up to 3000 ppm^45,46^.

Neutron capture radiotherapy based on ^10^B capture agents with external neutron beams is already an established radiotherapy modality, with traditional reactor-based neutron sources now being supplanted by more compact accelerator-based beam facilities. A number of accelerator-based epithermal neutron beam are in various stages of development and commissioning in hospitals in Japan, Finland, Russia, Taiwan, Argentina, Italy and the UK^47–56^. Two ^10^B delivery agents, L $-p-$ boronophenylalanine (^10^B-BPA) and sodium mercaptoundecahydro-closo-dodecaborate (Na_2_^10^B_12_H_11_SH; Na_2_^10^B-BSH) have been used clinically to treat patients suffering from glioblastoma multiforme and malignant melanoma, with Phase II and III clinical trials for the treatment of glioblastoma (GBM), head and neck tumours and liver metastases underway in Japan, Finland, Sweden, Japan, Taiwan, and the United States^57–65^. However, treatment of tissues deeper than approximately 3 cm is challenging with external epithermal neutron beams, due to the very high neutron fluence at the surface which is required to achieve a therapeutic effect at the target - a consequence of the neutron-moderating effect of the water in human tissue^66,67^.

In this paper, the quantity and spatial distribution of the thermal neutron fluence generated by typical therapeutic scanned-beam proton and carbon ion irradiations of homogeneous PMMA targets has been estimated via Monte Carlo simulations. The tissue concentrations of several ^10^B and ^157^Gd-based NCAs required to achieve an arbitrary 10% boost to the tumour biological effective dose are calculated, and compared to tissue concentrations in different tissues previously reported in the literature, with the aim of demonstrating the feasibility of NCEPT.

Details of the Monte Carlo simulations, including the primary beam energy and geometry, phantoms and physical models, as well as the method used in the quantification of the neutron capture dose enhancement are presented in Section 2. Neutron fluence results and the estimated neutron capture dose enhancement for boron and gadolinium is presented in Section 3, with the implications for enhanced biological effective dose discussed in Section 4. Conclusions and proposed future work are presented in Section 5.

## Materials and Methods

The method described here aims to establish whether NCEPT, when used in conjunction with a typical treatment plan, offers a significant therapeutic benefit compared to proton or heavy ion therapy alone. For the purpose of this study, significance is arbitrarily defined as an average 10% increase in photon-equivalent dose within the tumour resulting from the administration of a non-toxic bolus of neutron-capture agent; however, the method can be used with any desired dose increase factor. To answer this question, it is necessary to determine the concentration of neutron capture agent which is required in order to provide a 10% increase in effective photon-equivalent dose, for a simple simulated therapeutic proton/heavy ion treatment plan, and compare this with concentrations reported in the literature.

The first step is to evaluate the thermal neutron fluence (defined as neutrons with kinetic energy below 0.4 eV) resulting from pencil-beam irradiation of a point within a target volume. A set of simulations of this pencil beam, for both proton and ^12^C beams, is conducted with four different energies in a homogeneous PMMA target. Dose and thermal neutron fluence distributions are recorded for each simulation; corresponding distributions at energies in between these are also estimated by interpolating between the distributions obtained at these energies.

A simple treatment plan is then implemented, in which the pencil beam is stepped across an array of points inside a treatment volume at a series different energies. The primary particle fluence at each energy is then weighted such that a specified, approximately flat biological effective dose (BED) is delivered to a defined treatment volume by the ion beam. Two 50 mm cubic volumes are evaluated, once centred at a depth of 125 mm and a second centred at a depth of 165 mm. Thermal neutron fluence distributions throughout the entire phantom are then estimated based on the primary particle fluence weights for each treatment volume.

Based on the thermal neutron fluence estimates obtained through this process, the concentrations of a number of ^10^B and ^157^Gd-based NCAs required to achieve a 10% increase in biological effective dose is then determined for each treatment volume. The resulting NCA concentrations in normal tissue (defined as the total phantom outside of the treatment volume) are calculated using the reported tumour to normal tissue ratios for these NCAs. Maximum additional dose delivered to normal tissue is then determined as a percentage increase in dose to normal tissue in excess of the dose delivered by the primary beam.

### Simulation Parameters and Models

All Monte Carlo simulations were performed using the Geant4 toolkit (version 10.2.p03)^68,69^. Electromagnetic interactions were modelled using the standard Geant4 physics option 3 model (G4EmStandardPhysics_option3), while the hadronic physics models used in the simulations are listed in Table 1.

**Table 1:** Hadron physics models used in all simulations.

Interaction	Energy Range	Geant4 Model
Radioactive Decay	N/A	G4RadioactiveDecayPhysics
Particle Decay	N/A	G4Decay
Hadron Elastic	0–100 TeV	G4HadronElasticPhysicsHP
Ion Inelastic	0–110 MeV	Binary Light Ion Cascade
	100 MeV–10 GeV	QMDModel
	9.99 GeV–1 TeV	FTFP
Neutron Capture	0–20 MeV	NeutronHPCapture
	19.9 MeV–100 TeV	nRadCapture
Neutron Inelastic	0–20 MeV	NeutronHPInelastic
	19.9 MeV–9.9 GeV	Binary Cascade
Neutron Elastic	0 eV–20 MeV	NeutronHPElastic
	20 MeV–100 TeV	hElasticCHIPS
Proton Inelastic	0–9.9 GeV	Binary Cascade

A simple variance analysis method was used to determine the minimum number of primary particles to use in the simulations. A series of test simulations were conducted, each with *M* = 50 runs of $N(k)={2}^{k}{N}_{0},$ ${N}_{0}=1\times {10}^{5}$ primary particles. Thermal neutron fluence was calculated for each simulation within a test area centred on the Bragg peak, and the mean and standard deviation (SD) calculated across the *M* simulations. The inter-run standard deviation should approach zero as $N(k)$ tends to infinity; therefore, the experiment was repeated with progressively larger values of *k* until the ratio of inter-run standard deviation to mean was less than an arbitrary threshold of 5%. This analysis showed that *N* = 5 × 10^7^ incident protons and *N* = 5 × 10^6 12^C ions is sufficient to obtain a satisfactory estimate of thermal neutron fluence (99% probability of the estimated fluence being within ±5% of the true fluence).

### Pencil Beam Simulations

**Figure 1:**
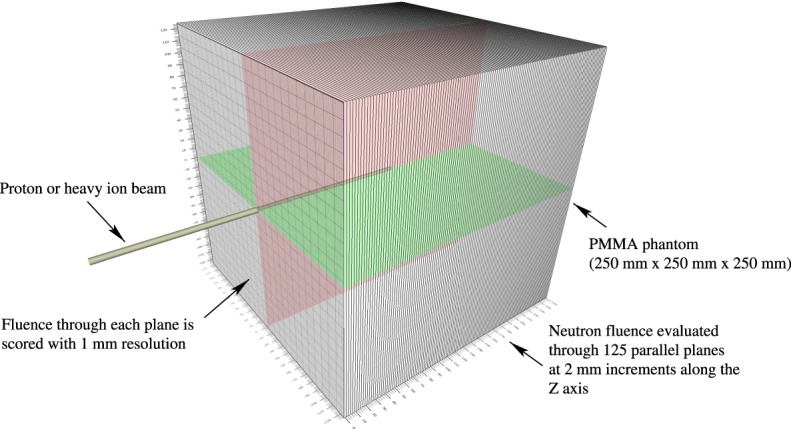
The simulation configuration used for pencil beam thermal neutron fluence estimation.

The Geant4 simulation and analysis configuration is shown in Fig. 1. Monoenergetic beams of protons and ^12^C ions with a rotationally symmetric 5 mm FWHM Gaussian beam profile were directed perpendicularly towards the surface of a homogeneous PMMA phantom. Four reference primary beam energies were chosen for the ^12^C beam, resulting in Bragg peak depths in PMMA of between 45 mm and 191 mm. Beam energies were then calculated for the proton beam such that the Bragg peaks were located at approximately the same depths. The full set of beam energies for each primary particle type and the corresponding locations of Bragg peaks in each phantom are listed in Table 2. The phantom is a homogeneous 250 mm × 250 mm × 250 mm cube of PMMA, with physical properties taken from the National Institute of Standards and Technology (NIST) database^70^. PMMA is chosen as the target material as it has a similar electron density and hence depth-dose profile to human tissue, and yields a similar range of fragmentation products despite its differing elemental composition^71^; moreover, it will be a convenient target for future experimental work.

**Table 2:** Primary energies of the proton and ^12^C beams applied to the PMMA phantom, and the depth of the point of maximum dose deposition (Bragg peak).

Particle	Energies (MeV/u)	Depths of Bragg Peaks (mm)
Proton ^10^C	73.0, 132, 153, 182	38.0, 109, 141, 191
	150, 250, 290, 350	45.0, 109, 140, 191

Pencil beam physical dose and thermal neutron fluence distributions were obtained for each beam type and energy, as listed in Table 2, and normalised per primary particle. To estimate dose and neutron fluence distributions for beam energies which were not simulated (due to the substantial computational cost of performing simulations for all intermediate energies), an interpolation procedure was performed. Firstly, the expected location of the Bragg peak for each intermediate energy was estimated via a 2nd-order polynomial interpolation between the locations measured from the dose distributions obtained at each of the four simulated energies. Next, the dose and thermal neutron fluence distributions from all but the highest-energy simulation were translated such that their Bragg peaks aligned with that of the highest energy simulation, and a 3D spatial interpolation of the the dose and neutron fluence distributions for the intermediate energies was performed. Finally, the interpolated 3D dose and neutron fluence distributions were translated back to the previously-estimated location of the Bragg peak for each energy. The result was a library of estimated physical dose distributions and thermal neutron fluence distributions per primary particle for proton and ^12^C beams, for energies in steps of 1 MeV/u in the range 73–182 MeV/u for protons and 150–350 MeV/u for ^12^C. While the method is only an approximation, its accuracy can be improved if desired by performing simulations at additional energies in the range of interest.

The library of physical doses distributions deposited by the pencil beams was then converted to biological dose; for protons, the relative biological effectiveness factor was assumed to be 1.1, while for ^12^C it was assumed to be 2.5, 3.0 and 3.3 for the pencil beams corresponding to the proximal, centre and the distal edges of the spread-out Bragg peak, 1.5 in the entrance plateau and buildup region (defined as the region with a deposited dose less than 60% of the maximum value), and a linear interpolation between these values in the intermediate regions^72–74^. The biological dose distributions were then used to develop a simple treatment plan for two target volumes for each beam type. These three-dimensional dose distributions for the centred pencil beam at the *k* th energy ($k\in \mathrm{[1}\ldots K]$) are denoted $BE{D}_{ctr,k}$. The corresponding neutron flux is denoted ${\varphi }_{ctr,k}$.

### Estimated Neutron Capture Dose Enhancement

As this study is concerned with determining the feasibility of NCEPT rather than evaluating a specific treatment plan, a set of simple treatment plans were developed for the PMMA target in to estimate the order of neutron capture agent concentration that would be required to achieve a 10% increase in photon-equivalent biological dose. For each energy, the BED and thermal neutron fluence maps (calculated via the interpolation method previously introduced) are stepped across the transverse (*xy*) plane of the treatment volume corresponding to the Bragg peak depth at each energy, through *R* row and *C* column positions (a total of *R* × *C* positions in the transverse plane) for each of the *k* energies:

$$BE{D}_{k}=\frac{1}{RC}\sum _{r=1}^{R}\sum _{c=1}^{C}\{BE{D}_{ctr,k}(r,c)\}$$

$${\varphi }_{k}=\frac{1}{RC}\sum _{r=1}^{R}\sum _{c=1}^{C}\{{\varphi }_{ctr,k}(r,c)\}$$

where $BE{D}_{ctr,k}(r,c)$ is $BE{D}_{ctr,k}$ laterally translated so that the centre of the Bragg peak is located at row and column $(r,c)$ in the plane, and ${\varphi }_{k}(r,c)$ is the corresponding neutron fluence. If the desired photon-equivalent dose is *D*, then the objective is to achieve the most uniform approximation of this dose possible within the treatment volume by determining the number of primary particles ${N}_{k}$ required at each energy $k$ which best approximate a flat biological effective dose. This is obtained by solving

$${{\rm{argmin}}}_{{N}_{k}}{\Vert (\sum _{k=1}^{K}{N}_{k}BE{D}_{k})-D\Vert }^{2}$$

using an optimisation technique such as Levenberg-Marquardt optimisation, subject to the constraint that *N*_*k*_ must be positive. The total number of primary particles required at each energy can then be multiplied by the corresponding map of neutron production per primary particle for each energy, to yield a map of total neutron fluence *ϕ* throughout the phantom (both inside and outside of the treatment volume):

$$\varphi =\sum _{k=1}^{K}\,{N}_{k}{\varphi }_{k}$$

The biological dose enhancement resulting from the presence of the neutron capture agent, normally referred to as the boron dose in BNCT literature, is estimated using the following relation^75^:

$${D}_{B}=\varphi {\sigma }_{NCA}{N}_{NCA}\times CBE$$

where ${\sigma }_{NCA}$ is the fluence-to-kerma conversion factor (approximately 8.66 × 10^−14^ for ^10^B and 9.27 × 10^−15^ for Gd^76,77^), *N*_*NCA*_ is the concentration of neutron capture agent in parts per million, and the the compound biological effectiveness *CBE* = 3.8 for ^10^B-BPA and ≈40 for the DOTA 157-Gadolinium triphenylphosphonium salt complex (based on results of studies in the field of photon activated therapy using the same agent, and correcting for expected Auger electron production)^76^. For this study, the target dose was set to *D* = 1 GyE, *R* = *C* = 11, and steps between rows and columns were set to 5 mm (i.e. the same as the FWHM of the beam) for a 50 mm square treatment plane at each energy. A range of energies were selected to extend the spread out Bragg peak (SOBP) between depths of 100 mm and 150 mm for the first treatment volume and 140 mm to 190 mm for the second; energies were incremented in steps of 1 MeV/u. Therefore, each treatment volume is a 50 mm cubic volume, with 1 GyE of dose delivered by the ion beam whilst the remainder of the phantom is regarded as normal tissue.

### Reported Neutron Capture Agent Concentrations

A selection of reported clinical and/or preclinical tissue concentrations of boron and gadolinium, together with the ratio of concentration in tumours to healthy tissue, are listed in Tables 3 and 4, respectively.

**Table 3:** Boron-based neutron capture agent concentrations and the ratios of tumour to healthy tissue concentrations reported in the literature.

Reported by	Method	Compound	Target	Concentration (PPM)	Tumour:normal ratio
Barth *et al*.^16^	Intravenous infusion	BPA	Brain	30 ± 12	5: 1
Luderer *et al*.^80^	Convection enhancement	BPA	Brain	68.3 ± 17.9	8: 1
Alkins *et al*.^23^	Ultrasonic enhancement	BPA	Brain	123 ± 25	6.7: 1
Suzuki *et al*.^78^	Inter-arterial infusion	BSH + lipidol	Liver	200 (6 h)	3.6: 1 (1 h), 14.9: 1 (6 h)
Suzuki *et al*.^78^	Inter-arterial infusion	BSH + degradable starch microspheres	Liver	231 (1 h)	1.4: 1 (1 h), 1.1: 1 (6 h)
Koganei *et al*.^25^	Intravenous infusion	BSH-encapsulating 10% DSBL liposomes	Colon	174 ± 20	1.2: 1–3.5: 1

**Table 4:** Gadolinium-based neutron capture agent concentrations reported in the literature. Tumour:normal tissue concentration ratios of at least 70 are commonly reported in the literature.

Reported by	Compound	Target	Concentration (PPM)
De Stasio *et al*.^38^	Gd-DOTA	GBM (*in vitro*)	140 (1 h)
Uyen *et al*.^100^	Gd-DTPA encapsulated liposome	TC-1 (mouse lung endothelium, *in vivo*)	159
Peters *et al*.^101^	Gd-DOTAP liposome	F98 & LN229 (glioma, *in vitro*)	768
Ichikawa *et al*.^102^	Gd-DTPA; Chitosan nanoparticles	B16F10 (mouse melanoma, *in vivo*)	1500
Tokumitsu *et al*.^103^	Gd-DTPA; Chitosan nanoparticles	B16F10 (mouse melanoma, *in vivo*)	1800
Morrison *et al*.^45^	Gd *III*-triarylphosphonium salts	T98G (glioblastoma, *in vitro*)	3000

## Results

### Treatment Plans and Neutron Fluence Distributions

**Figure 2:**
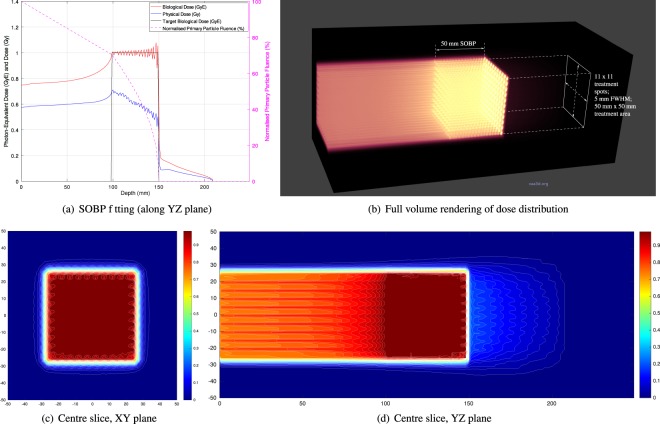
Photon-equivalent biological dose, physical dose and primary particle fluence (normalised to the total number of primary particles entering the phantom) resulting from 1 GyE carbon ion beam treatment of a 50 mm × 50 mm × 50 mm volume (100–150 mm depth); discrete beam energies range from 240–300 MeV/u in steps of 6 MeV/u). 2D slices and a 3D volume rendering of the dose distribution are also shown.

Treatment plans were prepared for each target volume for both proton and carbon ion beams. The total number of primary particles at each energy required for achieving an average biological dose of 1 GyE across the target volumes were computed, and the 3D dose distributions calculated. The case of carbon-ion irradiation of the shallower treatment volume (at depths ranging from 100 mm to 150 mm) is shown in Fig. 2.

**Figure 3:**
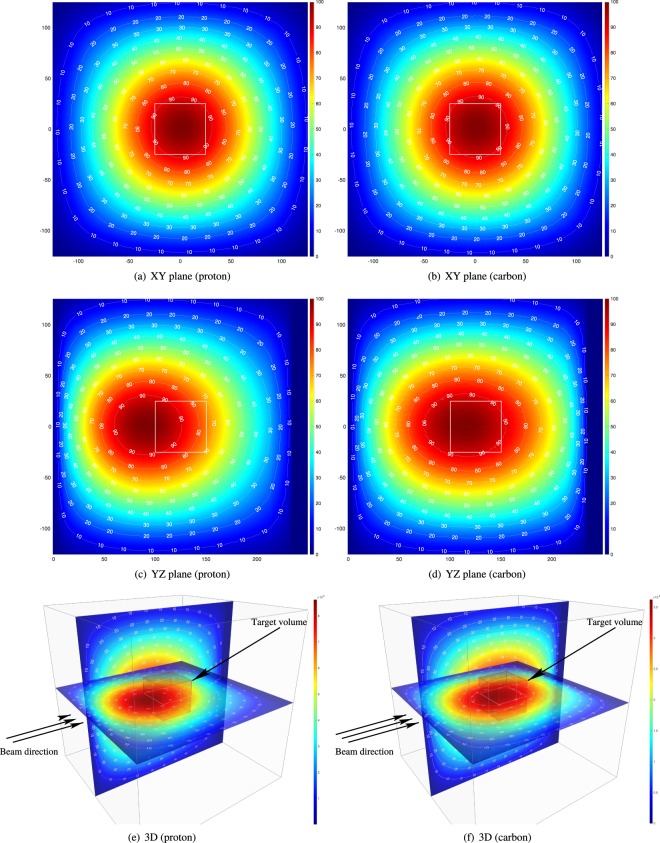
Normalised thermal neutron fluence resulting from irradiation of the 100 mm–150 mm target volume. Contour lines show fluence as a percentage of the maximum value in the slice, while the colourbars in the 3D figures show absolute fluence.

The per-primary-particle thermal neutron distributions corresponding to each of the energies in the treatment plan were scaled by the number of primary particles determined for each plan and summed for all energies required to form the spread out Bragg peak. An example of the resulting distribution of thermal neutron fluence (shown as a percentage of the maximum value on the contour maps and additionally as an absolute fluence in the 3D view) is shown in Fig. 3.

The maximum, mean and minimum thermal neutron fluences obtained within the treatment volumes are listed in Table 5.

**Table 5:** Thermal neutron fluences obtained for each target volume and treatment plan, assuming a target volume average proton or heavy ion biological dose of 1 GyE.

Target Depth (mm)	Primary Ion	Thermal neutron fluence per GyE primary dose (n/cm^2^/GyE)		
		Minimum	Mean	Maximum
100–150	Proton ^12^C	5.96 × 10^8^	7.79 × 10^8^	9.06 ×10^8^
		2.86 × 10^8^	3.34 × 10^8^	3.60 × 10^8^
140–190	Proton ^12^C	6.26 × 10^8^	8.82 × 10^8^	1.09 × 10^8^
		3.17 × 10^8^	4.08 × 10^8^	4.68 × 10^8^

### Required NCA Concentrations

The tumour concentrations of ^10^B and ^157^Gd required to achieve a 10% increase in biological effective dose are listed in Tables 6 and 7, respectively. The CBEs for each agent are based on values estimated in each listed supporting publication.

**Table 6:** ^10^B-based thermal neutron capture agent concentrations required to obtain a 10% increase in biological effective dose.

Target Depth (mm)	Primary	^10^B thermal neutron capture agent concentration (ppm)			
		BPA (brain)^24^	BSH (brain)^104^	BPA (liver)^26^	BSH (liver)^26^
		RBE = 3.8	RBE = 1.2	RBE = 9.94	RBE = 4.22
100–150	Proton ^12^C	390 910	1240 2880	149 348	351 820
140–190	Proton ^12^C	345 744	1090 2360	132 285	310 670

**Table 7:** ^157^Gd concentrations required to obtain a 10% increase in biological effective dose. Estimated values are based on published RBEs for non-specific multiple-Auger-electron-emitting nuclei. The right-most columns are based on Monte Carlo simulation results^31^.

Target Depth (mm)	Primary	^157^Gd thermal neutron capture agent concentration (ppm)				
		Cell^36^	DNA^36^	DNA^35^	MC: DNA^31^	MC: Cell^31^
		RBE = 5	RBE = 20	RBE = 10	RBE = 12.5	RBE = 1.5
100–150	Proton ^12^C	2790 6510	697 1630	1400 3260	1110 2580	9300 21700
140–190	Proton ^12^C	2460 5330	616 1330	1230 2660	978 2110	8220 17800

Normal tissue NCA concentrations are calculated on basis of the tumour concentrations required for a 10% increase in biological effective dose and the tumour:normal tissue concentration ratios reported in the literature. The maximum additional dose to normal tissue is then determined using this estimate of normal tissue NCA concentration together with normal-tissue CBEs reported in the literature and the maximum neutron fluence estimated in the normal tissue volume. The maximum percentage increases in dose to normal tissue resulting from a 10% increase in biological effective dose in both treatment volumes, are listed for all treatment volumes and all evaluated NCAs in Table 8.

**Table 8:** Maximum percentage increase in biological dose delivered to normal tissue, for a 10% increase in biological effective dose in the tumour.

Maximum increase in normal tissue biological dose (%)				
BPA (brain)^24^	BSH (brain)^104^	BPA (liver)^26^	BSH (liver)^26^	^157^Gd (all)
0.68	2.60	1.50	7.40	0.14

## Discussion

Several conclusions may be drawn by examining the tumour concentrations of each NCA listed in Tables 6 and 7. Firstly, the NCA concentrations required to achieve a 10% increase in biological effective dose in the liver are substantially lower than those required in the brain for both BPA and BSH, with BPA looking particularly promising due to the combination of high CBE and good tumour:normal tissue contrast reported by Suzuki *et al*.^26^. On the other hand, BSH concentrations have been reported in the literature which would realise a dose boost of close to 10% - for example, Suzuki *et al*.^78^ reported up to 200 and 234 ppm for BSH plus two different embolising agents, which would offer dose boosts of the order of 6.4–7.5% in the liver.

The situation is somewhat less positive for the brain; ^10^B-BPA concentrations required to achieve a 10% increase in biological effective dose during proton therapy in the brain would need to be around three times greater than the highest concentrations reported in the literature to date, while the concentration needed for carbon ion therapy is even greater. Conversely, with the highest BPA concentration reported in the literature of 125 ppm, the increase in dose is approximately 3.2–3.6% for proton therapy, and about half that for carbon. These results do not rule out the use of boron neutron capture agents for NCEPT treatment in the brain, but demonstrate the need for further development of boron-based NCAs.

Tantalisingly, there are reports in the literature of strong uptake of BPA in the pancreas, an organ in which cancer is notoriously difficult to treat^79^. While there appears to be very little research into BNCT specifically applied to the pancreas (particularly on tumour to normal NCA concentration ratios and CBE), it would appear to be a good candidate for NCEPT.

Several promising new ^10^B-based NCAs are still in development^80^. BSH has been somewhat disappointing as an NCA in BNCT, chiefly due to its inability to directly penetrate the cell membrane. However, several BSH-derived compounds have been proposed that combine up to 8 instances of the BSH compound with peptide chains, which are able to penetrate the membrane and deliver high concentrations of boron within the cell. Boron concentrations in excess of 5000 ppm have been reported for these compounds^81^. Other promising recent studies have investigated the use of using boron nitride nanotubes as NCAs in BNCT, which can also potentially deliver very high ^10^B concentrations to the tumour^82^.

For ^157^Gd, the situation is more complex. The values are highly dependent on how the ^157^Gd atoms are distributed; when they are either electrostatically attached to DNA or concentrated in the cell nucleus, the required concentrations are well within the ranges reported in the literature; this remains true even when the gadolinium is present in the cytoplasm or outside of the cell membrane. Several of the gadolinium compounds now in development appear to have many very promising properties for highly selective tumour uptake, and in particular high uptake in the nucleus and mitochondria, where they are most effective for neutron capture therapy. Significantly, many of the recently developed gadolinium-based compounds appear to offer very high tumour:normal tissue concentration ratios.

By comparing the required tumour concentrations obtained in this study to values previously published for both boron (up to 231 ppm in the liver^26^) and gadolinium (up to 3000 ppm *in vitro*^45^) it is clear that for certain existing combinations of NCA and target tissues, achieving an increase in biologically effective dose of at least 10% - or, equivalently, **reducing the primary radiation dose and hence reducing the radiation dose to normal tissue** - is feasible.

Additionally, there is also the possibility of further increasing the thermal neutron yield of heavy ion therapy. Since the production of neutrons within the target volume is typically considered a nuisance rather than a central objective, there has been little research aimed at identifying particular primary species which will result in greater rates of thermal neutron production in human tissue targets. We hypothesise that relatively neutron-rich primary ion species such as deuterium or helium may increase the thermal neutron yield, and therefore providing a larger dose boost via thermal neutron capture than is possible with either protons or carbon ions. This is currently a subject of further investigation, with results to be reported in future work.

Regarding the additional dose introduced to healthy tissues resulting from implementation of NCEPT, Table 8 shows that for most proposed NCAs, the increased dose is quite small compared to the dose boost delivered to the tumour (the worst-case scenario being BSH in the liver, due to the relatively low tumour:normal tissue contrast ratio of 0.3). For a 70 GyE primary ion dose to the tumour (typically delivered over several fractions), if the BPA concentration is sufficient to provide an extra 7 Gy tumour dose via NCEPT, the maximum additional normal-tissue dose (at the margin of the treatment volume) would be 0.47 GyE in the brain and 1.1 GyE in the liver (with 1.8 GyE and 5.2 GyE obtained with BSH in the brain and liver, respectively). For comparison, a BNCT treatment plan for glioblastoma multiforme typically delivers a peak dose of 8–14 GyE to normal brain tissue over 2–3 fractions^83^.

Intra-tumour and inter-patient tumour heterogeneity resulting in variable biodistribution and pharmacokinetics is a major problem in cancer treatments which rely on the targeted delivery of drugs to the malignancy, such as chemotherapy, therapeutic nuclear medicine and BNCT^84–88^. In clinical BNCT, theranostic approaches based on the use of radiolabelled or dual boron/gadolinium agents have been proposed to quantify the biodistribution of NCAs via positron emission tomography (PET) or magnetic resonance imaging (MRI) to improve the efficacy of neutron capture therapies^32,89,90^. ^18^F-BPA, a radio-labelled analogue of ^10^B-BPA has been successfully employed to determine the macrodistribution of boron in the tumour and estimate the tumour to normal tissue uptake ratio prior to treatment for treatment planning purposes^88,91–95^. A similar approach to quantify the biodistribution of the NCAs in target volume is necessary to optimise the treatment delivery in NCEPT.

With the additional dose provided through neutron capture, achievement of a prescribed uniform (or non-uniform) total radiation dose (ion plus neutron capture) throughout the tumour volume will require modifications to the treatment planning process. An initial plan can be constructed which only considers the primary ion dose. From this, an estimate of the thermal neutron field can be computed. Finally, either by assuming a flat distribution of NCA in the tumour, or by measuring it voxel-by-voxel using the theranostic approach mentioned previously, the estimated additional dose can be computed, after which the primary dose can be iteratively modulated until the total dose converges on the desired target dose prescription.

One possible criticism of the NCEPT approach is the need to fractionate the delivery of the therapeutic dose, which would either necessitate the use of a NCA with a long residence time or require repeated infusion of the NCA. However, the most recent literature recommends delivery of heavy ion radiotherapy via hypofractionation (1–2 fractions only)^6,96–98^. From a practical perspective, this makes addition of a boron-bearing drug infusion step to the treatment process a minimal additional burden on the patient, as it may only need to be performed once or twice.

As a final observation on the practicality of NCEPT: the main impediment to widespread adoption of neutron capture therapy is the limited availability of suitable epithermal neutron sources rather than the availability of appropriate NCAs^99^. NCEPT has the potential to offer a new source of thermal neutrons at any proton or heavy ion treatment facility, conveniently situated at the point of treatment inside the patient’s own body. With the prospect of further progress in the development of new NCAs, with greater tumour specificity and potentially very high achievable tumour concentrations, and possibly in combination with ultrasonic or other uptake enhancement methods, it may be possible to achieve even greater dose enhancement in the future.

## Conclusion

Our results demonstrate that the thermal neutron fluence distribution resulting from proton and carbon ion therapy mostly originates in the vicinity of the Bragg peak (i.e. from a point internal to the treatment volume), with the neutron fluence falling with increased distance from the Bragg peak in all directions. The fluence distribution resulting from a realistic treatment plan is sufficient to enable a significant increase of the order of 10% with realistic NCA concentrations of the order of magnitude previously reported in the literature. The resulting dose increase in normal tissues is quite modest, and is unlikely to cause additional harm to the patient. To our knowledge, this is the first time this concept has been proposed.

## Data Availability

The datasets generated and/or analysed during the current study are available from the corresponding author on reasonable request.
